# Mechanical ventilation modulates Toll-like receptor-3-induced lung inflammation via a MyD88-dependent, TLR4-independent pathway: a controlled animal study

**DOI:** 10.1186/1471-2466-10-57

**Published:** 2010-11-19

**Authors:** Carrie D Chun, W Conrad Liles, Charles W Frevert, Robb W Glenny, William A Altemeier

**Affiliations:** 1 Department of Medicine, University of Washington, 1959 NE Pacific St, Seattle, WA 98195, USA; 2 Department of Physiology and Biophysics, University of Washington, 1705 NE Pacific St, Seattle, WA 98195, USA; 3 Department of Comparative Medicine, University of Washington, 1959 NE Pacific St, Seattle, WA 98195, USA; 4 Department of Medicine, McLaughlin-Rotman Centre for Global Health, McLaughlin Centre for Molecular Medicine, Toronto General Research Institute, University of Toronto, Toronto, ON, M5G 2C4, Canada

## Abstract

**Background:**

Mechanical ventilation augments lung inflammation resulting from exposure to microbial products. The objective of this study was to test the hypothesis that ventilator-associated immune modulation requires MyD88-dependent signaling. Because MyD88 is a critical adapter protein utilized for pro-inflammatory signaling by all Toll-like receptors (TLRs), with the exception of TLR3, as well as by the IL-1 and IL-18 receptors, MyD88 dependence would implicate generation of an endogenous soluble ligand recognized by one or more of these receptors during mechanical ventilation and would provide an opportunity for a potential future therapeutic intervention.

**Methods:**

We compared the effect of mechanical ventilation on lung inflammation and permeability between poly(I:C) exposed mice with or without expression of MyD88. Poly(I:C) is a synthetic ligand for TLR3, the only MyD88-independent TLR, allowing isolation of the effect of MyD88 deletion on ventilator-augmentation of lung inflammation. Lung inflammation was assessed by cytokine concentration in lung tissue homogenate and polymorphonuclear cell (PMN) number in bronchoalveolar lavage fluid (BALF). Lung permeability was assessed by total protein, IgM, and intravenously injected FITC-dextran concentrations in BALF.

**Results:**

We found that MyD88 was required for mechanical ventilation augmentation of TLR3-induced lung inflammation and permeability. Because TLR4 is the most commonly reported receptor for endogenous ligands generated during tissue injury, we performed a second experiment comparing wildtype and TLR4-/- mice. We found that mechanical ventilation increased TLR3-mediated inflammation and permeability independent of TLR4.

**Conclusion:**

These data support the hypothesis that mechanical ventilation with moderate tidal volumes generates an endogenous ligand(s) recognized by MyD88-dependent receptor(s) other than TLR4, and that this mechanism can contribute to the development of ventilator-associated lung inflammation and injury. Identification of these ligands and/or receptors could lead to new pharmacological treatments for ARDS.

## Background

Acute lung injury (ALI) and its more severe presentation, the acute respiratory distress syndrome (ARDS), are important causes of mortality and health care expenditure in the United States and elsewhere. The estimated incidence of ALI in the United States is 196,000 cases annually with an estimated mortality of 38.5% [1]. Most patients with ARDS require mechanical ventilation, and multiple clinical studies have demonstrated that a lung protective ventilatory strategy employing lower tidal volumes alone or combined with end-expiratory pressure sufficient to prevent expiratory alveolar collapse reduces mortality in patients with ARDS [2-4].

Most clinical studies have focused on ventilator-associated lung injury (VALI) in the setting of pre-existing ARDS, but a retrospective case review by Gajic et al. reported that 24% of patients without clinical lung injury at the onset of mechanical ventilation eventually developed ALI and that the risk of developing ALI was positively correlated with the magnitude of tidal volume used during ventilation [5]. A second retrospective study by Jia et al. also identified tidal volume magnitude as an independent risk factor for the subsequent development of ALI in mechanically ventilated patients without pre-existing lung injury [6]. Studies using animal models support this finding. In rabbits, mechanical ventilation during endotoxemia synergistically increases alveolar polymorphonuclear cell (PMN) recruitment and cytokine expression in BALF beyond that seen with either endotoxin or mechanical ventilation alone [7,8]. In mice, mechanical ventilation modulates cytokine expression following both intra-tracheal lipopolysaccharide (LPS) instillation and intra-peritoneal LPS administration [9,10]. Mechanical ventilation augments PMN recruitment, cytokine expression, and lung permeability in murine models of *Staphylococcus aureus *pneumonia [11] and viral pneumonia [12]. Augmented lung inflammation and injury with mechanical ventilation also occurs with a variety of non-microbial insults such as hyperoxia [13,14] and intra-tracheal acid instillation [15]. Thus, these data suggest that ventilation with a strategy that does not independently cause clinically significant inflammation or injury may amplify the host response to pro-inflammatory stimuli, such as bacterial or viral infection, resulting in the development of acute lung injury.

How mechanical ventilation modulates lung inflammation and injury in response to microbial products or other inflammatory insults remains unknown. One possibility is that mechanical ventilation causes release of one or more endogenous ligands (i.e. signaling molecules generated by the host such as secreted cytokines or products resulting from tissue injury) for transmembrane receptors, associated with pro-inflammatory signaling. These ligands could be either classical cytokines, such as IL-1β, IL-18, TNFα, or Fas ligand (CD178), or other damage-associated molecular patterns (DAMPs) released during cellular injury and recognized by pattern recognition receptors such as the Toll-like receptors. Multiple DAMPs have been described for the cell surface TLRs 2 and 4 [16-18]. Mechanical ventilation with very large tidal volumes generates hyaluronan fragments recognized by TLR4 [19]; however, whether this occurs at lower tidal volumes is unknown. Additionally, there is increasing evidence that the intracellular TLRs 3, 7, 8, and 9 can also respond to endogenous nucleotides and may therefore play an important role in inflammation resulting from tissue damage [20-24].

Evaluating these multiple different receptors individually presents a significant challenge. However, an alternative strategy takes advantage of the fact that all of the TLRs, with the exception of TLR3, as well as many of the early response cytokine receptors utilize the adapter protein, myeloid differentiation factor 88 (MyD88) [25,26]. In contrast TLR3 requires the Toll/IL-1 receptor domain containing adapter inducing interferon (TRIF) adapter protein [27]. Unique among members of the TLR family, TLR4 can signal via both the MyD88 and TRIF adapter proteins [28,29].

**Figure 1:**
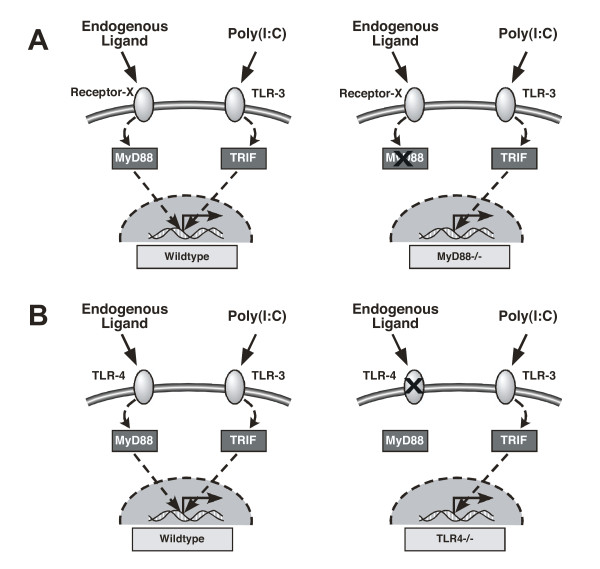
**Schematic of study strategy to test whether pro-inflammatory endogenous ligands are generated during mechanical ventilation**: (A) experiment#1 - evaluation of MyD88-dependent signaling during mechanical ventilation and TLR3 activation in WT and MyD88-/- mice; (B) experiment#2 - evaluation of TLR4-dependent signaling during mechanical ventilation and TLR3 activation in WT and TLR4-/- mice.

We hypothesized that mechanical ventilation with conventional tidal volumes releases one or more endogenous ligand(s) recognized by a MyD88-dependent receptor(s) and that the resulting intracellular signal augments the inflammatory response to concurrent exposure to exogenous or microbial TLR ligands. By combining a TLR3-specific ligand and mice lacking functional MyD88, we were able to evaluate whether mechanical ventilation modulated inflammatory responses via a MyD88-dependent mechanism (Figure 1A). We found that mechanical ventilation with conventional tidal volumes modulates inflammation and lung permeability via MyD88-dependent pathways. Because TLR4 is the most commonly implicated MyD88-dependent receptor for damage-associated endogenous ligands, we evaluated the role of TLR4-dependent signaling during mechanical ventilation by measuring the inflammatory response of wild-type (WT) and TLR4-/- mice to a TLR3 ligand (Figure 1B). However, mechanical ventilation with moderate tidal volumes did not require TLR4 for augmentation of inflammation.

## Methods

### Reagents and Mice

The TLR3 specific ligand polyinosinic:polycytidylic acid (poly(I:C)) was purchased from Invivogen, diluted to a concentration of 10 mg/mL in sterile pyrogen-free PBS (Gibco), and stored in small aliquots at -20°C. Aliquots were thawed immediately before each experiment. Reagents for multiplex bead immunoassays were purchased from Millipore (Lincoplex).

The University of Washington Office of Animal Welfare approved these experiments. MyD88-/- (C57Bl/6 background) and TLR-4-/- (C57Bl/6 background) mice originally generated by Shizuo Akira (Osaka University) were obtained from Thomas Hawn (University of Washington) and Chris Wilson (University of Washington), respectively. Control C57Bl/6 mice were purchased from Jackson Laboratories. All mice were housed in a specific-pathogen free facility. MyD88-/- and TLR-4-/- mice were bred at the University of Washington and genotype confirmed by PCR followed by gel electrophoresis. Additionally, phenotype was confirmed in select littermates by hypo-responsiveness to Pam_3_CysSerLys4, a synthetic TLR2 ligand (MyD88-/-), or lipopolysaccharide (TLR-4-/-). Mice between 8 and 14 weeks of age were used for the experiments.

### Experimental protocol

All mice were anesthetized with 5% isoflurane for 5 min, suspended by the front teeth at a 60° angle, and intra-tracheally inoculated with 2 μL per gram body weight of poly(I:C) as previously described [9]. All mice were then returned to their cages.

30 min after instillation, mice were assigned to either mechanical ventilation or spontaneous respiration. Mice assigned to mechanical ventilation were anesthetized with ketamine 0.66 mg and xylazine 0.04 mg i.p. injection and 5% isoflurane for 5 min and suspended by the front teeth at a 60° angle to allow direct visualization of the vocal cords by external laryngeal trans-illumination. Mice were orally intubated with a 20-gauge angiocatheter (BD Biosciences) cut to 30 mm length with a beveled tip. The catheter was positioned to a depth of 20 mm at the teeth and secured with a drop of glue. Mice were connected to a volume-cycled mouse ventilator (Minivent; Harvard Biosciences) with the following settings: tidal volume of 10 mL/kg body weight, respiratory rate of 150 breaths per minute, positive end-expiratory pressure of 3 cm H_2_O, and FiO_2 _0.21. Mice received a 500 μL subcutaneous injection with dextrose 5% lactated Ringer's solution (D5LR; Baxter) at the start of mechanical ventilation, followed by continuous subcutaneous infusion of D5LR at 250 μL/hr. Anesthesia and neuromuscular blockade were achieved with continuous inhaled 1% isoflurane and pancuronium 10 μg/hr i.p., respectively. Three hours after the start of mechanical ventilation, mice were injected with 100 μL of 14 mM 70-kD fluorescein isothyocyanate dextran (FITC-D; Invitrogen) in sterile water into the retro-orbital vascular sinus for subsequent determination of lung permeability. Parameters measured continuously during mechanical ventilation included: airway pressure, temperature (via rectal thermistor) and mixed expiratory CO_2 _(Novametrix). Blood pressure and heart rate were measured every 30 min (SC-1000, Hatteras Instruments). At the end of 6 hr of mechanical ventilation, mice were euthanized under isoflurane anesthesia by cardiac puncture and exsanguination.

Mice assigned to spontaneous ventilation remained in their cages with access to food and water for 6 hours. Three hours prior to the end of the experiment, each mouse was anesthetized with 5% isoflurane for 5 min in order to undergo retro-orbital injection with 100 μL of 14 mM FITC-D. Mice were returned to their cages for the remainder of the experiment. After 6 hours of spontaneous ventilation, mice were sedated with ketamine (0.66 mg) and xylazine (0.04 mg) i.p. and 5% isoflurane for 5 min and then euthanized by cardiac puncture and exsanguination.

### Lung Homogenate and Bronchoalveolar Lavage Fluid (BALF) Preparation and Analysis

After euthanasia, the left lung was removed, weighed, and homogenized in 1 mL sterile water with protease inhibitor cocktail (cOmplete mini EDTA-free, Roche Diagnostics). Lung homogenate was vortexed with 32 μL of a 20× cytokine lysis buffer (final concentration: 0.5% Triton X-100, 150 mM NaCl, 15 mM Tris, 1 mM CaCl, 1 mM MgCl, pH 7.40). After 30 min incubation at 4°C and 20 min centrifugation at 10,000-g, the supernatant was stored in aliquots at -80°C for subsequent cytokine determination. Multiplex bead immunoassay (Lincoplex) was performed according to the manufacturer's protocol on the Luminex 100 platform to measure concentrations of KC/CXCL1, MCP-1/CCL2, MIP-1α/CCL3, IL-6, and TNF-α.

Bronchoalveolar lavage of the right lung was performed using three 0.5 mL aliquots of PBS containing 0.6 mM EDTA. A portion of the pooled BALF was set aside for total cell count by hemacytometer and for differential cell count using a cytospin preparation with Wright stain. The remaining BALF was centrifuged at 1500-g and 4°C for 10 min. BALF supernatant was removed and FITC-D concentration was determined by fluorescence spectroscopy at an excitation wavelength of 494 nm and emission wavelength of 521 nm (Perkin Elmer LS-50B). The fluid was then stored in small aliquots at -80°C for subsequent determination of total protein concentration by Bradford Assay (Pierce Biotechnology) and IgM concentration by ELISA (Bethyl Laboratories).

### Statistical Analysis

Data were analyzed by two-way ANOVA, using presence or absence of mechanical ventilation and genotype as the main effects. Additionally, our model included an interaction term to determine whether the effect of mechanical ventilation was dependent on genotype. Post-hoc comparisons, using a Bonferroni correction, were performed between genotypes for both ventilation conditions if either the genotype term or the mechanical ventilation · genotype interaction term were significant. Airway pressures were compared between the mechanically ventilated groups over time, using a repeated measures ANOVA. Post-hoc comparisons for each time point were done using a Bonferroni correction. Statistical significance was assigned at p ≤ 0.05. All analyses were performed using Prism software (Graphpad). Data are presented as mean ± standard error of the mean (SEM).

## Results

In the first experiment evaluating MyD88, 42 male mice were studied. Results from 40 mice are presented (10 per group). Two mice were excluded from analyses because of errors in FITC injection. In the second experiment evaluating TLR4, 40 mice of both genders were studied. Six male and 4 female mice were included in each experimental group. Results from all 40 mice are presented.

### Role of MyD88 in ventilator-associated augmentation of TLR3-induced lung inflammation

**Figure 2:**
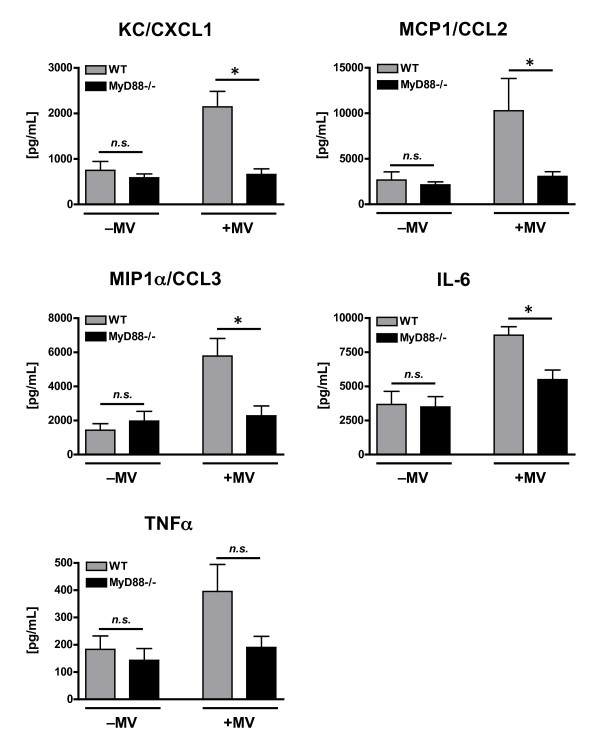
**Cytokine concentrations in lung homogenate from WT and MyD88-/- mice treated with intra-tracheal poly(I:C), a TLR3 agonist, in the presence or absence of concurrent mechanical ventilation (MV)**. *n = 10/group*. ⁎ - p < 0.05, comparing different genotypes within the same mechanical ventilation condition.

Overall, there were significant effects from both mechanical ventilation and genotype on expression of KC/CXCL1, MCP1/CCL2, MIP1α/CCL3, and IL-6 in poly(I:C) treated mice (Figure 2). A significant interaction effect between mechanical ventilation and genotype, indicating that mechanical ventilation affected the cytokine response to poly(I:C) differently depending on genotype, was observed for expression of KC/CXCL1 and MIP1α/CCL3. A trend towards an interaction between mechanical ventilation and genotype was observed for IL-6 (p = 0.056) and MCP-1/CCL2 (p = 0.080).

There were no observed differences in any of the cytokine levels between WT and MyD88-/- mice treated with poly(I:C) in the absence of mechanical ventilation (Figure 2). This was anticipated as TLR3 is a TRIF-dependent, MyD88-independent receptor and confirms that there were no contaminating exposure to ligands, which would be recognized by a MyD88-dependent receptor (e.g. LPS). In contrast, the lungs of mechanically ventilated, poly(I:C)-treated WT mice had significantly greater levels of KC/CXCL1 (2143 ± 340 vs. 657 ± 125 pg/mL), MCP1/CCL2 (10,283 ± 3554 vs. 3063 ± 513 pg/mL), MIP1α/CCL3 (5775 ± 1033 vs. 2280 ± 575 pg/mL), and IL-6 (8750 ± 624 vs. 5497 ± 696 pg/mL) as compared to the lungs of similarly treated MyD88-/- mice (Figure 2). Additionally, there was a non-significant trend towards more TNFα in ventilated WT mice as compared with MyD88-/- mice (396 ± 98.7 vs. 191 ± 40.5 pg/mL, p = 0.07). These data indicate that augmentation of poly(I:C)-induced cytokine response during mechanical ventilation occurs primarily via a MyD88-dependent pathway.

**Figure 3:**
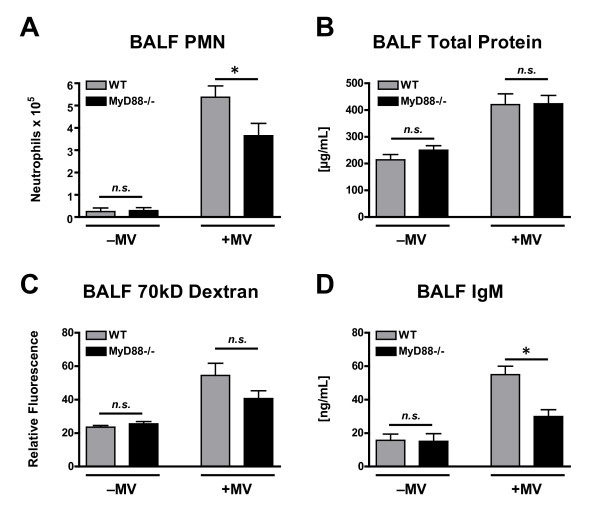
**Polymorphonuclear cell (PMN) count (A), total protein concentration (B), 70-kD FITC-dextran concentration (C), and IgM concentration (D) in the BALF of WT and MyD88-/- mice treated with intra-tracheal poly(I:C), a TLR3 agonist, in the presence or absence of concurrent mechanical ventilation (MV)**. *n = 10/group*. ⁎ - p < 0.05 comparing different genotypes within the same mechanical ventilation condition.

Similar to lung cytokine measurements, there was an overall significant effect of both mechanical ventilation and genotype on BALF PMN count in poly(I:C)-treated mice (Figure 3A). Additionally, there was a strong interaction effect, indicating that the effect of mechanical ventilation on BALF PMN count was genotype dependent. Consistent with this, there were more PMNs in the BALF of mechanically ventilated WT mice as compared with mechanically ventilated MyD88-/- mice (5.4 ± 0.5 × 10^5 ^vs. 3.6 ± 0.6 × 10^5 ^PMN, p < 0.05, Figure 3A). There was no difference in BALF PMN count between non-ventilated WT and MyD88-/- mice exposed to poly(I:C).

Mechanical ventilation increased both BALF total protein (Figure 3B) and 70 kD FITC-D (Figure 3C) concentrations in mice exposed to poly(I:C). There was no effect of genotype and no mechanical ventilation · genotype interaction on either total protein or 70 kD FITC-D concentration in the BALF; although, there was a non-significant trend towards a mechanical ventilation · genotype interaction effect on 70 kD FITC-D BALF concentration (p = 0.081, Figure 3C). In contrast to total protein and FITC-D, there were significant effects of both mechanical ventilation and genotype on BALF concentration of IgM (Figure 3D). A strong interaction effect between genotype and mechanical ventilation was present for BALF IgM concentration, resulting in a significant difference between mechanically ventilated WT and MyD88-/- mice (55.0 ± 5.1 vs. 29.0 ± 4.0 ng/mL) but not between spontaneously breathing WT and MyD88-/- mice (Figure 3D). Because IgM exists primarily as a pentameric molecule with a molecular weight of ~900-kD, these data suggest that MyD88-dependent signaling contributes to ventilator-associated disruption of the alveolar-capillary barrier sufficient to allow leakage of large plasma proteins into the airspace. Interestingly, in contrast to cytokine production, both PMN recruitment and vascular permeability resulting from the combination of mechanical ventilation and poly(I:C) treatment were only partially attenuated in MyD88-/- mice, suggesting the presence of MyD88-independent pathways by which mechanical ventilation modulates the host response toTLR3 activation.

**Figure 4:**
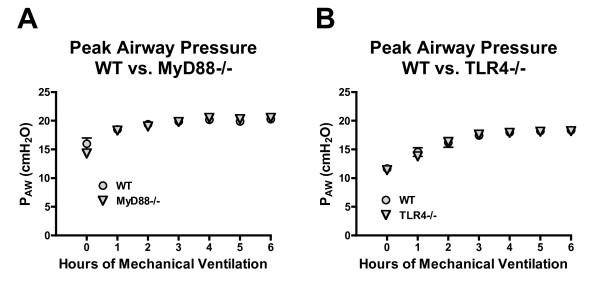
**Peak airway pressures in mechanically ventilated, poly(I:C)-treated WT and MyD88-/- mice (A) and WT and TLR4-/- mice (B)**. *n = 10/group*.

Peak airway pressures significantly increased over time during mechanical ventilation for both WT and MyD88-/- poly(I:C)-treated mice. However, there was no effect of genotype on peak airway pressures and no differences were observed between genotypes at any time (Figure 4A).

To summarize the first set of experiments, mechanical ventilation amplified cytokine expression following TLR3 stimulation via MyD88-dependent pathway(s). Both MyD88-dependent and MyD88-independent pathways were required for maximal PMN recruitment and vascular permeability resulting from the combination of mechanical ventilation and poly(I:C) treatment.

### Role of TLR4 in ventilator-associated augmentation of TLR3-induced lung inflammation

The first set of experiments demonstrated that mechanical ventilation augments TLR3-induced cytokine production, PMN recruitment, and lung permeability via one or more MyD88-dependent pathways. MyD88 is an adapter protein, linking transmembrane receptors involved in innate immune signaling and subsequent pro-inflammatory responses. Therefore, these data suggest that mechanical ventilation causes release of endogenous ligand(s) recognized by one or more MyD88-dependent receptors. TLR4 is a frequently identified receptor for tissue damage-associated endogenous ligands, particularly in the setting of sterile tissue reperfusion injury, and is involved in reperfusion injury to the lung [30], heart [31,32], liver [33,34], kidney [35], and brain [36]. Additionally, TLR4 was recently reported to contribute to inflammation in the setting of low tidal volume ventilation [37]. We therefore tested whether mechanical ventilation with normal tidal volumes augmented inflammatory responses via a TLR4-dependent mechanism. To test this hypothesis, we compared poly(I:C)-treated WT and TLR4-/- mice randomized to either spontaneous breathing or mechanical ventilation.

**Figure 5:**
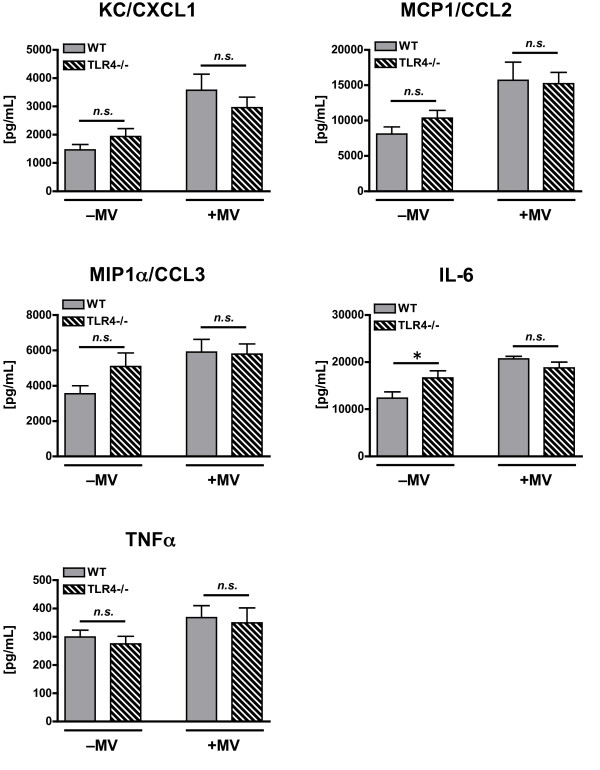
**Cytokine concentrations in lung homogenate from WT and TLR4-/- mice treated with intra-tracheal poly(I:C), a TLR3 agonist, in the presence or absence of concurrent mechanical ventilation (MV)**. *n = 10/group*. ⁎ - p < 0.05 comparing different genotypes within the same mechanical ventilation condition.

Consistent with the first experiment, there was a significant effect of mechanical ventilation on lung levels of KC/CXCL1, MCP1/CCL2, MIP1α/CCL3, and IL-6 in poly(I:C)-treated mice (Figure 5). A similar though non-significant trend was observed for TNFα (p = 0.056, Figure 5). In contrast to the first experiment there was no genotype effect on cytokine expression and no interaction effect between mechanical ventilation and genotype for any cytokine with the exception of IL-6. This interaction effect for IL-6 was associated with significantly greater IL-6 in unventilated TLR4-/- mice as compared with unventilated WT mice. There was no difference in IL-6 between the ventilated, poly(I:C)-treated mice (Figure 5).

**Figure 6:**
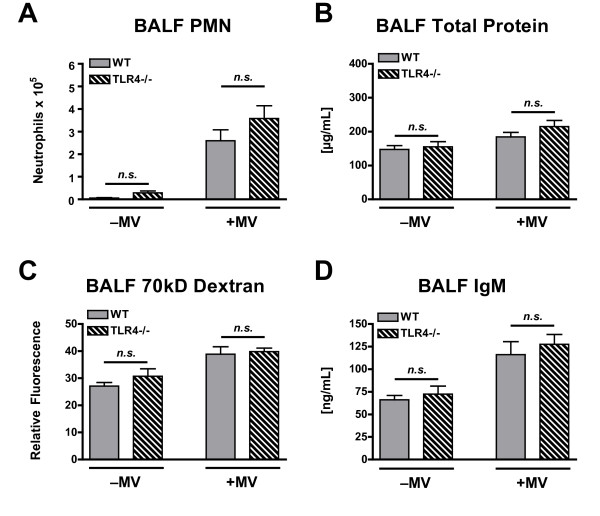
**Polymorphonuclear cell (PMN) count (A), total protein concentration (B), 70-kD FITC-dextran concentration (C), and IgM concentration (D) in the BALF of WT and TLR4-/- mice treated with intra-tracheal poly(I:C), a TLR3 agonist, in the presence or absence of concurrent mechanical ventilation (MV)**. *n = 10/group*. ⁎ - p < 0.05 compared with same genotype, spontaneously breathing.

There was a significant effect of mechanical ventilation on BALF PMN counts in both WT and TLR4-/- poly(I:C)-treated mice, and there were no differences between WT and TLR4-/- mice under either spontaneous breathing or mechanical ventilation conditions (Figure 6A). There was a significant mechanical ventilation effect on lung permeability of poly(I:C)-treated mice as measured by BALF total protein, 70 kD FITC-D, and IgM concentrations (Figure 6B-D). There were no significant effects from genotype or from a genotype · mechanical ventilation interaction for any of these permeability measurements.

Peak airway pressures significantly increased over time during mechanical ventilation for both WT and TLR4-/- poly(I:C)-treated mice. However, there was no effect of genotype on peak airway pressures and no differences were observed between genotypes at any time (Figure 4B).

In summary, this set of experiments yielded results similar to those observed in the first set of experiments in which mechanical ventilation augmented inflammation and lung permeability in the setting of TLR3 activation. There were no significant differences between WT and TLR4-/- mice responses to mechanical ventilation. These data show that mechanical ventilation does not require TLR4 to augment lung inflammation and injury associated with TLR3 activation.

## Discussion

Prior studies demonstrated that mechanical ventilation alone, using tidal volumes of 10 mL/kg, does not independently cause significant cytokine production, neutrophil recruitment, or lung permeability in mice. However, mechanical ventilation does augment both lung inflammation and injury in the presence of a variety of pro-inflammatory stimuli, including LPS, bacterial pneumonia, and viral pneumonia [9-12]. We hypothesized that mechanical ventilation with a tidal volume of 10 mL/kg generates endogenous ligands, either classical cytokines and/or damage-associated molecular patterns (DAMPs), which are recognized by MyD88-dependent transmembrane receptors, resulting in amplification of the inflammatory response from concurrently administered poly(I:C), a TLR3 ligand. Because all known TLRs except for TLR3, as well as many early response cytokine receptors, signal via the MyD88 adapter protein, we studied the effect of mechanical ventilation in the setting of TLR3 activation in normal (WT, MyD88+/+) mice and in MyD88-/- mice. The main findings of this study were: 1) mechanical ventilation augmented cytokine expression, PMN recruitment, and lung permeability, during TLR3 activation; and 2) maximal cytokine response, PMN recruitment, and vascular permeability induced by mechanical ventilation required the MyD88 adapter protein.

Because TLR4 signals via MyD88 and has been implicated as a primary receptor for DAMPs, we tested whether mechanical ventilation at normal tidal volumes resulted in TLR4-dependent signaling. The role of TLR4 in ventilator-associated lung injury is uncertain with conflicting data in the literature. Held and co-authors previously reported that high tidal volume ventilation causes NF-κB nuclear translocation and cytokine expression in lungs isolated from C3H/HeJ mice, which have a genetic polymorphism, resulting in a non-functional TLR4 [38]. In contrast, Vaneker and co-authors reported that low-tidal volume ventilation caused inflammation in wildtype but not in TLR4-/- mice [37]. Other studies have looked at the role of TLR4 in mechanical ventilation; however, these studies have also used concurrent LPS administration or bacterial infection, making it difficult to separate out the contribution of mechanical ventilation on TLR4-dependent signaling [39,40]. We found that mechanically ventilated, TLR4-/- mice concurrently exposed to the TLR3 ligand, poly(I:C), did not have significantly different cytokine concentrations, PMN recruitment, or lung permeability as compared to similarly treated WT mice. Our data support the work of Held et al that mechanical ventilation with normal tidal volumes does not generate endogenous ligands recognized by TLR4. The explanation for the different finding by Vaneker and colleagues is unclear but may relate to inadvertent LPS exposure during animal preparation or possibly an unanticipated interaction effect between the mechanical ventilation and the elevated inspired oxygen fraction (0.4) used in this study. Indeed interaction effects between ventilation and hyperoxia on inflammatory responses have been previously reported in animal models [13,14].

Selective attenuation of the mechanical ventilation effect on cytokine expression, PMN recruitment, and lung permeability in MyD88-/- mice suggests a role for signaling via the MyD88 adapter protein in ventilator-associated lung injury. The most likely mechanism involves stretch-induced generation of endogenous ligands, which signal via MyD88-dependent receptors. Known MyD88-dependent receptors include all of the TLRs with the exception of TLR3 [25], the IL-1 receptor [41], the IL-18 receptor [42], and Fas (CD95) [43]. There are no published data examining the effect of moderate tidal volume ventilation on signaling by these receptors; however, there is one report of increased IL-1α and IL-1β release induced by low tidal volume ventilation [44]. Identification of the MyD88-dependent pathways by which mechanical ventilation modulates innate immune responses could provide therapeutic avenues to reduce the risk of acute lung injury in mechanically ventilated patients; however, a thorough examination of these different pathways is beyond the scope of the current work.

Interestingly, mechanical ventilation is reported to increase expression of TLR4 and its associated co-adapter, CD14 [39,40,45,46]. Thus, upregulation of TLRs is one potential mechanism by which mechanical ventilation could modify inflammatory responses. Although we did not specifically measure TLR3 expression in the current study, review of our previously published microarray study indicates that TLR4 mRNA but not TLR3 mRNA is increased in mice mechanically ventilated with a tidal volume of 10 mL/kg as compared with control mice [9]. Therefore, upregulation of TLRs is unlikely to be the only mechanism through which mechanical ventilation can amplify inflammation during concurrent exposure to TLR ligands.

An important additional observation from these experiments was that ventilator-induced augmentation of PMN recruitment and lung permeability was only partially attenuated in MyD88-/- mice, indicating the presence of parallel MyD88-independent pathways. One possible explanation for this result is stretch-induced changes in endothelial cells. High tidal volume ventilation increases lung endothelial cell P-selectin and focal adhesion molecule expression [47]. These changes may promote vascular PMN demargination independent of TLRs and their respective adapter proteins. Another possibility is that the combination of mechanical ventilation and TLR3 activation causes cellular injury sufficient to release mitochondrial-derived, formylated proteins, which have recently been reported as a mechanism for neutrophil recruitment in the setting of sterile tissue injury [24].

## Conclusion

In summary, mechanical ventilation with tidal volumes of 10 mL/kg augmented inflammation and lung permeability in response to TLR3 activation. Mechanical ventilation exerted its effects primarily via a MyD88-dependent but TLR4-independent mechanism. Because MyD88 functions as an adapter protein for transmembrane receptors, this study supports a role for receptor recognition of endogenous ligands in the pathogenesis of ventilator-associated lung injury. Further investigation to identify the relevant ligands and associated receptors will provide critical insight into designing new interventions to limit lung injury associated with mechanical ventilation.

## Competing interests

The authors declare that they have no competing interests.

## Authors' contributions

CDC performed all experiments, and participated in data analysis and manuscript preparation. WCL, CWF, and RWG contributed to experimental design, data interpretation, and manuscript preparation. WAA oversaw all experiments, data analysis, and manuscript preparation. All authors read and approved the final manuscript.

## Grant Support

This research was supported by National Institutes of Health Grants HL086883, HL73996, HL07287 and Canada Research Chair in Infectious Diseases and Inflammation (WCL)

## Pre-publication history

The pre-publication history for this paper can be accessed here:

http://www.biomedcentral.com/1471-2466/10/57/prepub
